# Activity of TNT: a phase 2 study using talimogene laherparepvec, nivolumab and trabectedin for previously treated patients with advanced sarcomas (NCT# 03886311)

**DOI:** 10.3389/fonc.2023.1116937

**Published:** 2023-05-10

**Authors:** Sant P. Chawla, Walter Andree Tellez, Hripsime Chomoyan, Chrysler Valencia, Amir Ahari, Nadezhda Omelchenko, Stefan Makrievski, Don A. Brigham, Victoria Chua-Alcala, Doris Quon, Ania Moradkhani, Erlinda M. Gordon

**Affiliations:** ^1^ Medical Oncology, Sarcoma Oncology Research Center, Santa Monica, CA, United States; ^2^ Gene and Cell Therapy, Aveni Foundation, Santa Monica, CA, United States

**Keywords:** talimogene laherparepvec, nivolumab, trabectedin, sarcoma, immunotherapy, alkylating agents

## Abstract

**Background:**

Intratumoral injection of talimogene laherparepvec evokes a cytotoxic immune response. Therefore, the combination of talimogene laherparepvec with trabectedin and nivolumab may have synergistic effects in advanced sarcomas.

**Patients and methods:**

This phase 2 trial was conducted from May 30, 2019 to January 31, 2022. Endpoints: Primary: Progression free survival rate at month 12. Secondary: Best overall response, progression free survival rate at 6 and 9 months, overall survival rate at 6, 9, and 12 months, incidence of conversion of an unresectable tumor to a resectable tumor, and incidence of adverse events. Eligible patients had to be ≥ 18 years of age, have advanced histologically proven sarcoma, at least 1 previous chemotherapy regimen, and at least one accessible tumor for intratumoral injection. Treatment: Trabectedin intravenously (1.2 mg/m^2^ q3 weeks), nivolumab intravenously (3 mg/kg q2 weeks), and intratumoral talimogene laherparepvec (1x10^8^ plaque forming units/ml q2 weeks).

**Results:**

Median time of follow-up: 15.2 months. Efficacy analysis: Thirty-nine patients who had completed at least one treatment cycle and had a follow-up computerized tomography were evaluable for efficacy analysis. Median number of prior therapies: 4 (range 1-11). Progression free survival rate at month 12, 36.7%. Confirmed Best Overall Response by Response Evaluation Criteria in Solid Tumors v1.1 = 3 partial responses, 30 stable disease, 6 progressive disease. Best Overall Response Rate, 7.7%, Disease Control Rate, 84.6%; median progression free survival, 7.8 (95% Confidence Intervals: 4.1-13.1) months; 6-, 9-, 12-month progression free survival rates, 54.5%/45.9%/36.7%; median overall survival 19.3 (95% Confidence Intervals: 12.8 -.) months; 6-, 9- and 12-month overall survival rate, 86.9%/73.3%/73.3%. One patient had a complete surgical resection. Fifty percent of patients had a ≥ grade 3 treatment related adverse events which included anemia (6%), thrombocytopenia (6%), neutropenia (4%), increased alanine transaminase (4%), decreased left ventricular ejection fraction (4%), dehydration (4%), hyponatremia (4%).

**Conclusions:**

Taken together these data suggest that the TNT regimen is effective and safe for advanced previously treated sarcomas, and is worth being further studied in a randomized phase 3 trial as first- or second- line treatment for patients with advanced sarcomas.

## 1 Introduction

According to SEER, 5-year survival rate for advanced metastatic sarcomas is only 15% (1). Therefore, innovative therapies are urgently needed. Advances in sarcoma therapy include the introduction of targeted therapies (inhibitors of the tyrosine kinase and serine threonine kinase signaling pathways), monoclonal antibodies against immune checkpoints (ipilimumab, nivolumab, pembrolizumab, avelumab, durvalumab and atezolizumab), virotherapy, tumor-targeted therapies (nab-paclitaxel), and molecular targeting of specific genetic mutations (ROS1, ALK, TRK1-3 (2–13) and CAR-T cell therapy (14).

Specifically, the goal of the combination regimen is to improve treatment outcome parameters including a two-fold prolongation of progression free survival (PFS) compared to the reported PFS of trabectedin alone (4.1 months, 15). Talimogene laherparepvec (TVEC), trabectedin and nivolumab are cancer drugs approved by the United States Food and Drug Administration (USFDA) with different mechanisms of action, efficacy and toxicity profiles. TVEC is a genetically modified oncolytic virus designed to replicate within tumors and to produce the immune stimulatory protein GM-CSF. Talimogene laherparepvec causes lysis of tumors, followed by release of tumor- derived antigens, which together with virally derived granulocyte macrophage-colony stimulating factor (GM-CSF) may promote an antitumor immune response (9). Nivolumab is a human IgG4 kappa monoclonal antibody that blocks the interaction between PD-1 and its ligands, PD-L1 and PD-L2 (7). Trabectedin is a marine derived alkaloid that simultaneously kills cancer cells and affects several features of the tumor microenvironment (TME), most notably by inducing the rapid and selective apoptosis of monocytes and macrophages, and by inhibiting the transcription of several inflammatory mediators. Furthermore, depletion of tumor associated macrophages (TAMs) alleviates the immunosuppressive milieu and rescues T cell functional activities, thus enhancing the anti-tumor response to immunotherapy with checkpoint inhibitors (10, 16). Retrospective analysis using a combination regimen of trabectedin and nivolumab for advanced soft tissue sarcomas (STS) showed a median PFS of 10 months (range: 10->95 weeks), median overall survival (OS) of 15 months, 6 month PFS rate of 68.2%, and 6 month OS rate of 95.4% (17).

We hypothesized that talimogene laherparepvec, an oncolytic HSV expressing human granulocyte macrophage colony stimulating factor (GMCSF), will have an additive effect to the drug combination, trabectedin and nivolumab, by inducing local oncolysis which would expose tumor neoantigens in the TME, by evoking macrophage maturation to dendritic cells, differentiation of M2 growth promoting macrophages to M1 killer macrophages which will then recruit CD8 killer T cells into the TME for tumor antigen recognition and restoration of normal tumor surveillance function.

## 2 Patients and methods

### 2.1 Patient eligibility

Eligible patients had to be ≥ 18 years of age, had to have metastatic or locally advanced histologically proven sarcomas with evidence of disease progression within the past month, had at least 1 previous chemotherapy regimen, had at least one accessible tumor for intratumoral injection of talimogene laherparepvec and at least one 1 cm target lesion for evaluation of antitumor activity using Response Evaluation Criteria for Solid Tumors Version 1.1 (RECIST v1.1; 18), an Eastern Cooperative Oncology Group (ECOG) score of 0-1 and adequate hematologic and organ function. Patients who had a history of an immune disorder, active central nervous system involvement, active herpetic skin lesions, acute or chronic hepatitis, acquired immune deficiency, pregnant or refused barrier contraception were excluded from participation.

### 2.2 Clinical trial design and regulatory oversight

This single center, open-label single-arm, phase 2 trial was conducted from May 30, 2019 to January 31, 2022. Treatment schedule: Trabectedin (1.2 mg/m^2^ q3 weeks), nivolumab (3 mg/kg q2 weeks), and TVEC (1x10^8^ PFU/ml q2 weeks) were administered. A starting dose of TVEC (1x10^6^ PFU/ml) was initially given, followed 3 weeks later by a dose of 1x10^8^ PFU/ml q2 weeks depending on tumor size three weeks later. Follow up computerized tomography (CT) or magnetic resonance imaging (MRI) were conducted within 30 days of enrollment and every 6 weeks thereafter. Per protocol, patients who did not complete one treatment cycle and had a follow up CT scan or MRI were replaced.

The study was approved by a Central Institutional Review Board (Western Institutional Review Board [WIRB], Olympia, WA) and conducted in accordance with institutional and federal guidelines for human investigation in accordance with the Declaration of Helsinki. All participants were informed of the investigational nature of the study and provided written informed consent prior to enrollment. This trial was sponsored by Amgen. Ongoing safety oversight was conducted by the WIRB. Any serious unexpected adverse events were reported to the WIRB and the U.S. Food and Drug Administration.

### 2.3 Endpoints

The primary end point was PFS rate at month 12 per RECIST 1.1 (18). Secondary endpoints were best overall response (BOR) by RECIST v1.1 *via* computerized tomography (CT) or magnetic resonance imaging (MRI), median PFS, PFS rate at 6 and 9 months, median OS, OS rate at 6, 9, and 12 months, incidence of conversion of an unresectable tumor to a resectable tumor, and incidence of adverse events related to trabectedin, nivolumab and talimogene laherparepvec. Exploratory endpoint was correlation of response with immune cell trafficking in the tumor microenvironment of resected tumors.

### 2.4 Statistical analysis

#### 2.4.1 Baseline descriptive statistics

Demographics, age at enrollment (<65 vs ≥65 and <75 vs ≥75 years), sex, subtypes of sarcoma, first- second- or third-line therapy, number of patients, patients with locally advanced or metastatic, resectable or unresectable tumors, and European Cooperative Oncology Group (ECOG) score were described using descriptive statistics.

#### 2.4.2 Definition of intention-to-treat and modified intention-to-treat

The Intention-to Treat (ITT) population consisted of all subjects, who received at least one dose of trabectedin, nivolumab or talimogene laherparepvec. The modified Intention-to-Treat (mITT) population was composed of all patients who had completed the first cycle of trabectedin, nivolumab and talimogene laherparepvec and had a CT scan or MRI at the 6-week follow-up period. Patients who did not complete one treatment cycle and did not have a follow-up CT scan or MRI were replaced. The ITT population was used for analysis of overall survival and incidence of adverse events. The mITT population was used for analysis of the primary and secondary end points.

#### 2.4.3 Analysis of primary endpoint

PFS at 12 months was assessed by RECIST v1.1 (18) with the data from CT/MRI.

#### 2.4.4 Analysis of secondary endpoints

Response was assessed utilizing RECISTv1.1 (18). Computed tomography (CT) scans were compared against baseline imaging every 6 weeks. Responses were classified as confirmed complete response (CR), partial response (PR), stable disease (SD), or progressive disease (PD). Survival curves for PFS and OS were estimated by the Kaplan-Meier method (19). The number of patients at risk and the number of patients censored were provided. Median PFS and median OS was estimated, along with its two-sided 95% confidence interval (20, 21). A two-fold prolongation of median progression free survival or better in evaluable patients served as a milestone for moving forward with a Phase 2/3 randomized study.

#### 2.4.5 Safety analysis

Incidence and severity of adverse events and significant laboratory abnormalities were performed on all patients (ITT population). Patient incidence of all treatment related adverse events (TRAEs) were tabulated by system organ class and preferred term using National Cancer Institute Common Toxicity Criteria for Adverse Events Version 5 (22). Tables of fatal adverse events, serious adverse events, TRAEs, and adverse events leading to withdrawal from investigation product were provided. For trabectedin, nivolumab and talimogene laherparepvec, exposure summary statistics were provided for total number of doses.

#### 2.4.6 Analysis of exploratory endpoints

The analyses of the exploratory endpoints were descriptive in nature.

#### 2.4.7 Sample size and justification

The number of patients studied were 39 evaluable patients in the mITT group. Patients who withdrew or did not complete the first 6-week cycle and first follow up CT scan/MRI were replaced. Although this study was not large enough to allow firm conclusions about safety or efficacy, it provided preliminary data on safety and efficacy that would be useful in planning future Phase 2 studies. A two-fold prolongation of median progression free survival in evaluable patients compared to median PFS reported with trabectedin alone (4.1 months, 15) was determined as a milestone for moving forward with Phase 2/3 randomized study.

## 3 Results

### 3.1 Patient characteristics

**Table 1** shows the baseline characteristics of patients enrolled according to age group, sex, histologic subtypes, and number of previous cancer therapy regimens. Twenty-two of 50 patients received a doxorubicin-based regimen as first line therapy, followed by either enrollment in a clinical trial or an FDA approved drug for advanced sarcomas. Five of 50 patients received gemcitabine and docetaxel as first line therapy. One patient with endometrial carcinosarcoma, an undifferentiated sarcoma, was included in the study, after she failed standard therapy for endometrial carcinoma. Her initial treatment included radical hysterectomy and adjuvant therapy with 6 cycles of carboplatin + paclitaxel plus 4,500 cGy delivered to the pelvis over 25 fractions. She developed metastatic disease 1 ½ years later, was treated with liposomal doxorubicin with disease progression. She was then enrolled in the TNT protocol, had stable disease and clinical benefit for 15 months, and was alive as of the data cutoff date.

**Table 1:** Baseline characteristics.

Patients	n = 50
Age	
18-28	4 (8%)
29-39	12 (24%)
40-50	7 (14%)
51-61	12 (24%)
62-72	9 (18%)
73-83	6 (12%)
Sex	
Men	23 (46%)
Women	27 (54%)
ECOG Score	
< 1	50 (100%)
Histological type	
Undifferentiated Pleomorphic Sarcoma	3 (6%)
Spindle cell sarcoma	2 (4%)
Leiomyosarcoma, uterine = 8 (16%)	15 (30%)
Leiomyosarcoma, Non-uterine = 7 (14%)	
Sacral chordoma	1 (2%)
Osteosarcoma	4 (8%)
Ewing sarcoma	2 (4%)
Clear cell sarcoma	1 (2%)
Extraskeletal myxoid chondrosarcoma	1 (2%)
Synovial sarcoma	8 (16%)
Desmoplastic small round cell tumor	1 (2%)
Epithelioid sarcoma	1 (2%)
Rhabdomyosarcoma	1 (2%)
Liposarcoma, dedifferentiated = 4 (8%)	8 (16%)
Liposarcoma, myxoid = 4 (8%)	
Carcinosarcoma	1 (2%)
Myoepithelioma	1(2%)
Number of chemotherapy regimens	
Intention-to-Treat: median (range)	4 (range: 1-11)
Modified Intention-to-Treat: median (range)	3 (range: 1-11)

### 3.2 Summary of efficacy analysis

The median time of follow up for patients included in the efficacy analysis was 15.2 months (Interquartile Range: 6.67 to 24.14). The cut-off point for PFS and OS was 1/31/2022. Eleven patients were not deemed evaluable for efficacy analysis because they did not complete the first treatment cycles and did not get a follow-up CT scan. Thirty-nine patients were evaluable for efficacy analysis.

As shown in **Table 2.0**, for the mITT population (n=39), the PFS rate at 12 months was 36.7%. There were 3PR, 30SD, 6PD; Best Overall Response Rate (BORR) 7.7%, Disease Control Rate 84.6%. Median PFS was 7.8 (95%CI: 4.1-13.1) months, Median OS, 19.3 (95%CI: 12.8 -.) months; 6/9/12- month PFS Rates were 54.5%/45.9%/36.7% and 6/9/12-month OS Rates, 86.9%/73.3%/73.3%. As shown in **Table 2.1**, for the ITT population, median OS was 15.8 (95%CI: 6.4-23.4), 6/9/12- month OS rate, 69.6%/58.7%/58.7%. The best responders (PR) were patients with uterine leiomyosarcoma (LMS), dedifferentiated liposarcoma (LPS) and undifferentiated pleomorphic sarcoma (UPS).

**Table 2.0:** Efficacy analysis: best overall response, progression free survival, overall survival for the modified intent-to-treat population (n = 39).

Best Response RECIST v1.1	Disease Control Rate, %	Median PFS Months (Range)	Median OS Months (Range)	6/9/12 month PFS Rate %	6/9/12- month OS Rate %
3PR, 29SD*,	84.6%	7.8	19.3	54.5/45.9/36.7	86.9/73.3/73.3
6PD		(1-25)	(9-32)		
BORR 7.7%		(95% CI: 4.1-13.1)	(95% CI:12.8 -)		

*One patient with SD had a surgical CR.

**Table 2.1:** Overall survival for the intent-to-treat population (n=50).

Median OS months (range) (CI)	6/9/12-month OS rate %
15.8	69.6/58.7/58.7
(0-32)	
(95% CI: 6.4-23.4)	

**Figure 1:**
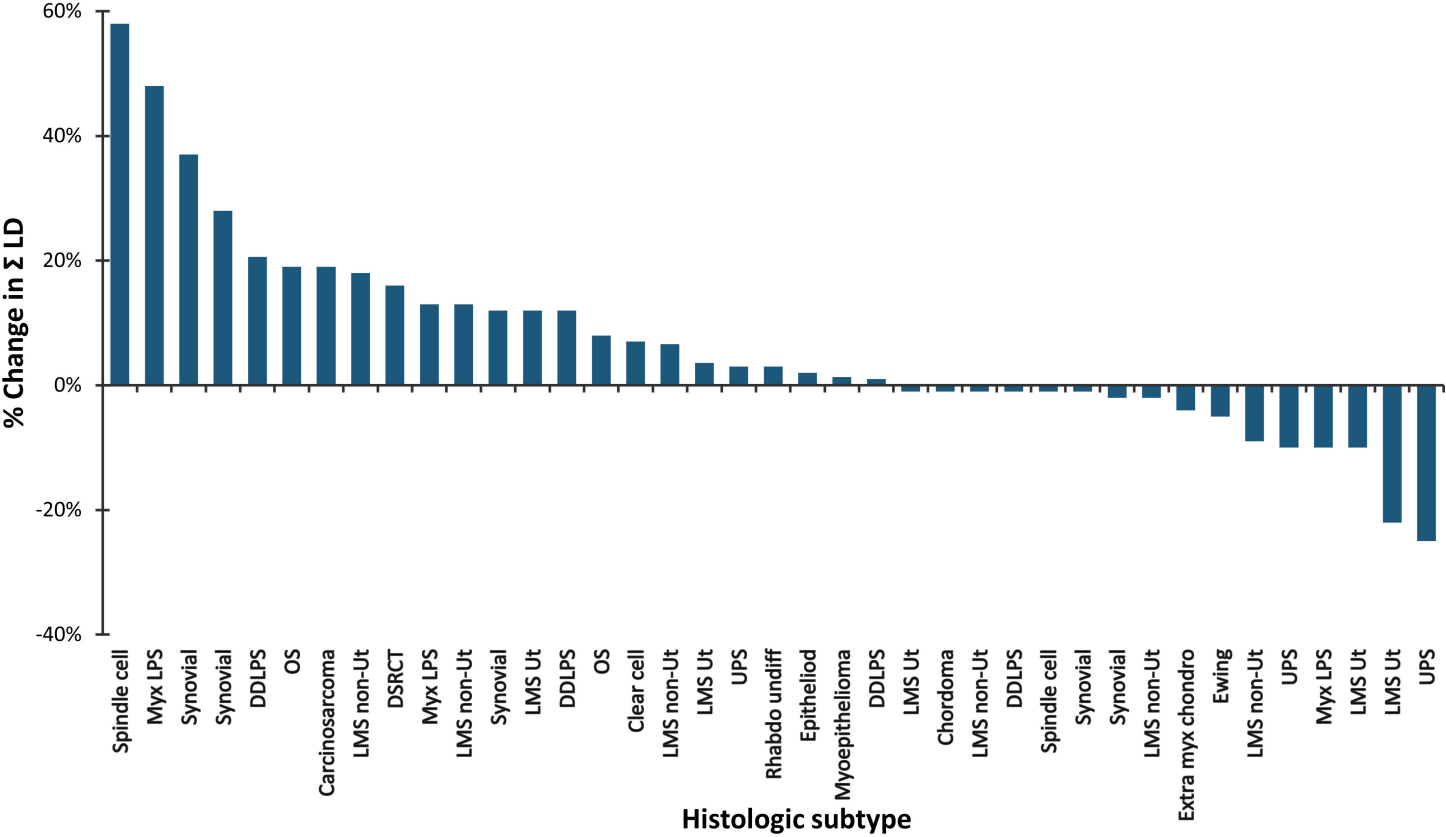
Water Fall plot of percent change in the sum of the longest diameters by RECIST v1.1 (n = 39). Percent change at Week 6 is plotted on the vertical axis and histologic subtype is plotted on the horizontal axis.

**Figure 2:**
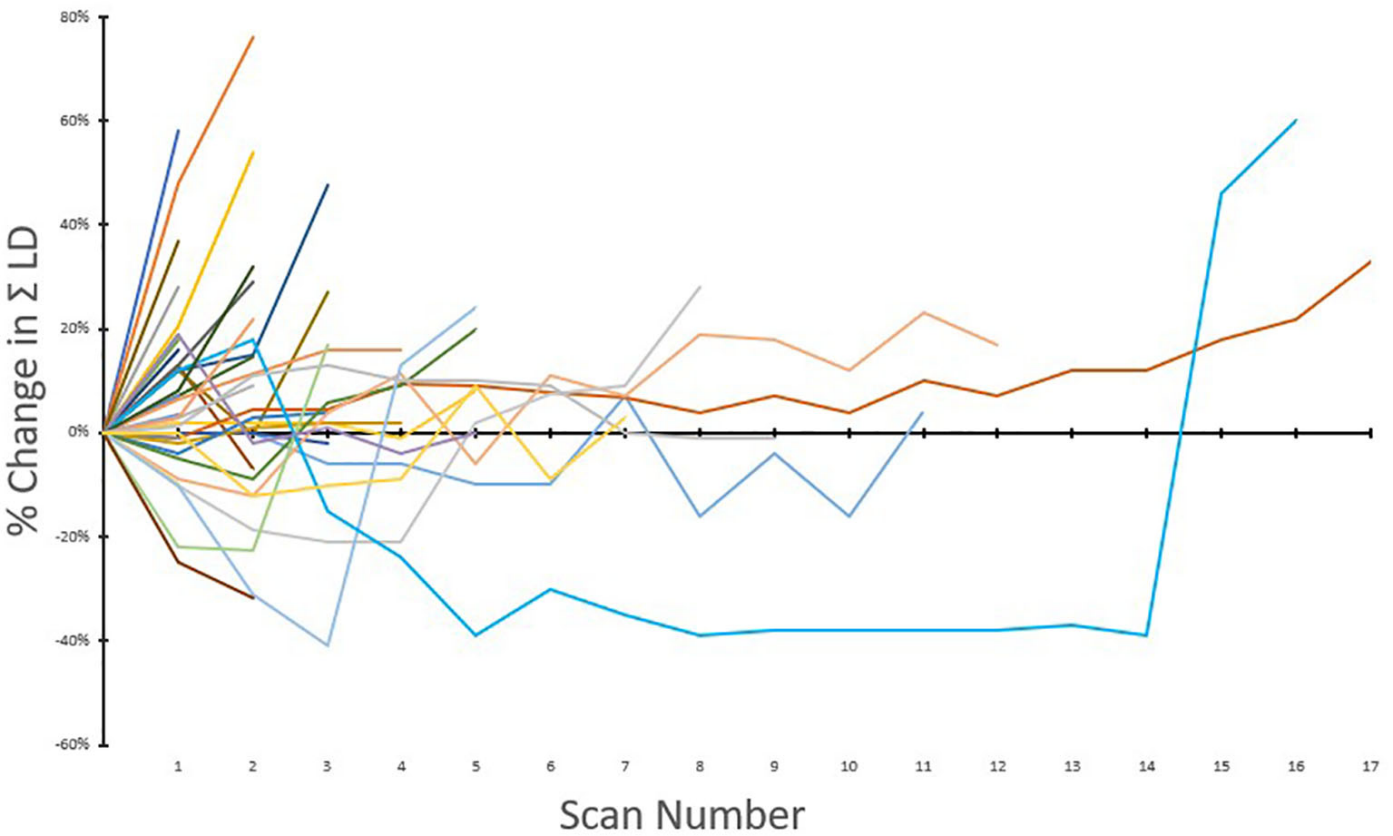
Spider plot of percent change in the sum of longest diameters with scan number (n = 39). Percent change is plotted on the vertical axis as a function of scan numbers.

**Figure 3:**
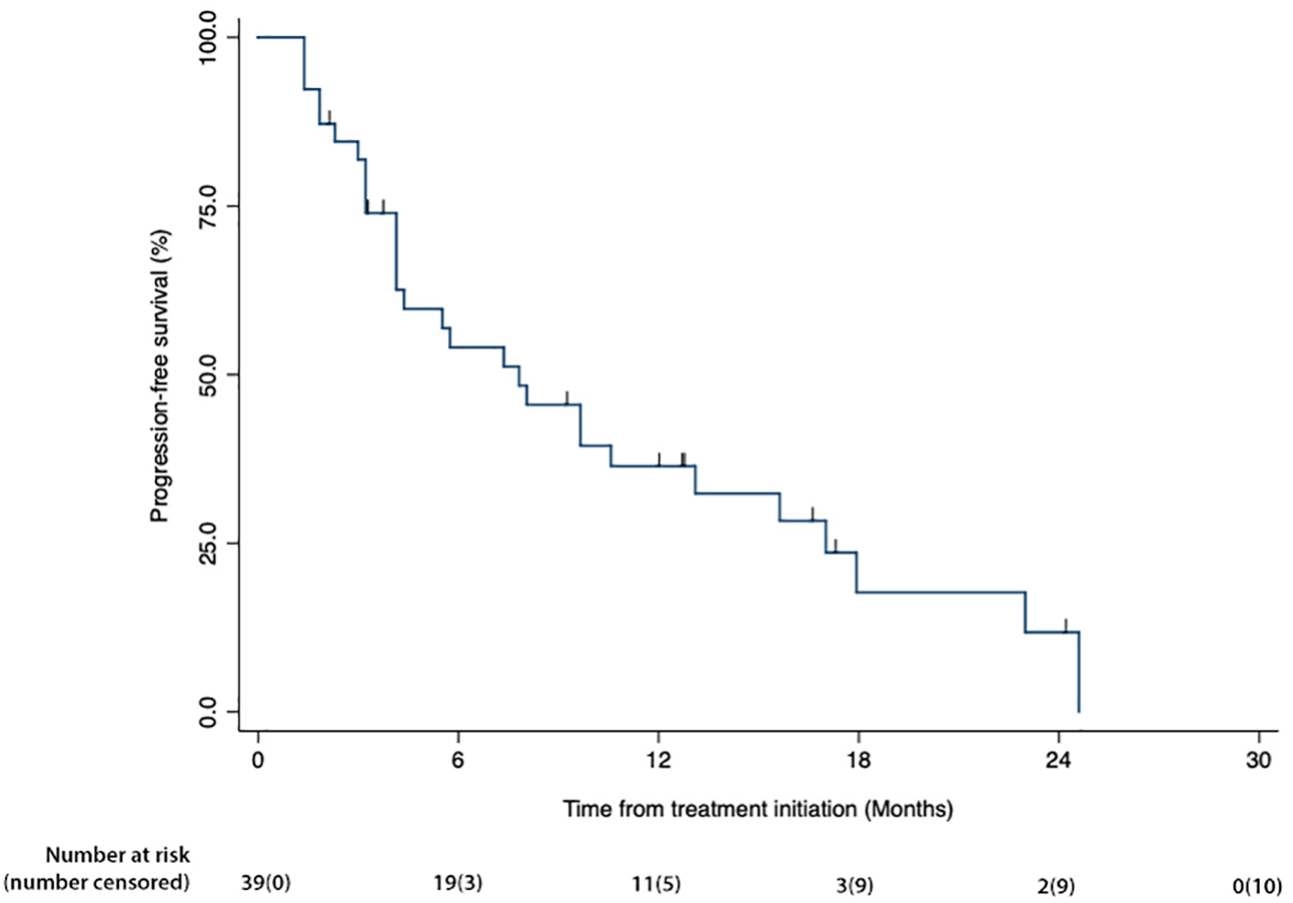
Kaplan Meier plot of progression-free survival for the mITT group. Percent surviving is plotted on the vertical axis as a function of time (months), plotted on the horizontal axis.

**Figure 4:**
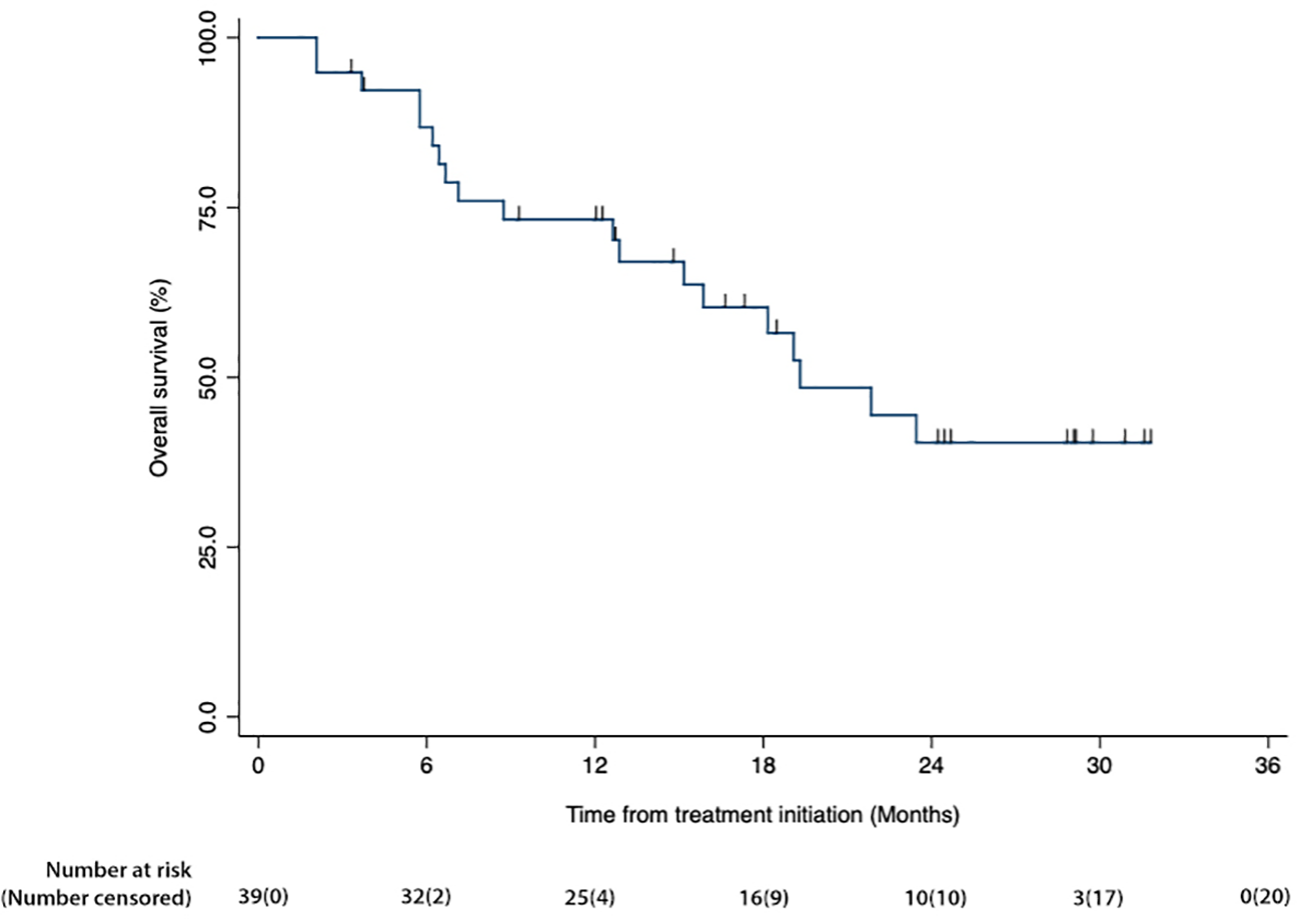
Kaplan Meier plots of overall survival for the mITT group. Percent surviving is plotted on the vertical axis as a function of time (months), plotted on the horizontal axis.

**Figure 1** is a waterfall graph of percent change in sum of target lesions longest diameter (SLD) at week 6 for each patient and histologic subtype, **Figure 2** shows the spider plot of percent change in SLD with each CT scan. For 3 partial responders, it took 3-5 treatment cycles for them to achieve their best response (partial response). For the rest, week 6 was the best response time. **Figure 3** shows the Kaplan Meier plot for progression free survival and **Figure 4** shows the Kaplan Meier plot for overall survival.

### 3.3 Survival at data cut-off

As of the Data Cut-off, 15 patients are known to be alive; of these, one patient (carcinosarcoma) is continuing nivolumab after completing one year of therapy and one patient (myoepithelioma) is continuing talimogene laherparepvec and nivolumab after completing one year of therapy. One patient with locally advanced rhabdomyosarcoma received intratumoral injections of talimogene laherparepvec, followed by surgical resection. She is in sustained remission 2 years s/p surgical resection on no further cancer therapy.

As of data cut-off, 29 patients have died from disease progression, 6 patients withdrew due an adverse event and 6 patients were lost to follow-up. Supplementary materials are provided for all adverse events (A, B), and listings of deaths (C), patients who withdrew (D) and who were lost to follow up (E).

### 3.4 Exposure to treatment

**Table 3** lists treatment exposure data: the number of trabectedin (T), nivolumab (N) and talimogene laherparepvec (Tv) infusions for each patient. Trabectedin was discontinued in patients with unacceptable toxicity related to trabectedin. Trabectedin was discontinued after one year of therapy according to protocol. Nivolumab was continued beyond one year on a case by case basis outside of protocol in patients who had stable disease or better and no unacceptable toxicity related to nivolumab.

**Table 3:** Treatment exposure.

Patient Number	Number of Infusions (T/N/TVEC)
1	4/6/6
2	3/4/3
3	12/28/23
4	4/7/5
5	14/17/15
6	2/2/2
7	5/9/7
8	6/15/14
9	3/4/3
10	3/3/2
11	9/19/16
12	4/5/3
13	6/10/4
14	15/42/42
15	11/14/9
16	6/7/6
17	4/5/3
18	19/33/26
19	9/12/11
20	2/3/2
21	6/15/12
22	2/3/2
23	3/3/2
24	1/12/11
25	2/2/2
26	11/17/11
27	16/49/44
28	5/6/5
29	2/2/1
30	6/4/4
31	13/38/38
32	18/31/26
33	5/5/5
34	6/7/2
35	2/1/1
36	3/5/3
37	14/19/17
38	4/4/2
39	14/15/10

Number of infusions of trabectedin, nivolumab and talimogene laherparepvec (TVEC) per patient.

### 3.5 Summary of safety analysis

Safety was assessed by the incidence of treatment related and unrelated adverse events (AE) according to the National Cancer Institute (NCI) Common Terminology Criteria for Adverse Events (CTCAE) version 5.0 (22).

Twenty-five of 50 (50%) ITT patients had a = grade 3 TRAE. **Table 4** shows ≥ Grade 3 treatment- related adverse events according to drug and severity grade.

**Table 4:** Grade 3 or greater adverse events related to study therapy by causality.

Adverse Events	Related to trabectedin		Related to nivolumab		Related to talimogene laherparepvec	
	3	4	3	4	3	4
Blood and lymphatic system disorders						
Anemia	8 (16%)					
General disorders and administration site conditions						
Fatigue	2 (4%)					
Pain at tumor site					1 (2%)	
Investigations						
Alanine aminotransferase increased	9 (18%)					
Aspartate aminotransferase increased	3 (6%)					
Neutrophil count decreased	7 (14%)		1 (2%)			
Platelet count decreased	4 (8%)	1 (2%)				
CPK increased	1 (2%)					
GGT increased	1 (2%)					
Ejection fraction decreased	1 (2%)					
T3 decreased			1 (2%)			
Metabolism and nutrition disorders						
Dehydration	1 (2%)					
Hyponatremia			1 (2%)			

### 3.6 Correlative analysis

**Figure 5:**
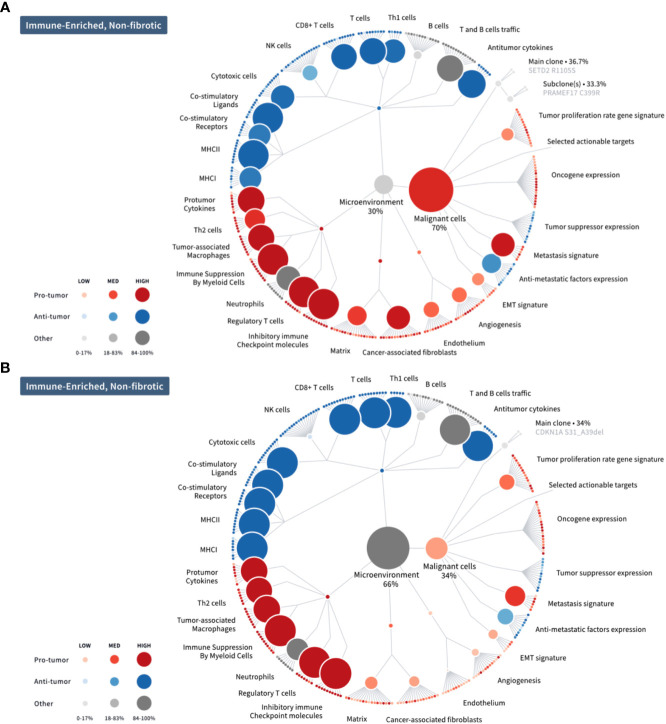
Molecular functional portraits of pre and post TNT treatment tumors from a patient with advanced rhabdomyosarcoma. **(A)** Before treatment with TNT; **(B)** After treatment with TNT. Comparative evaluation of the molecular functional portraits of pre and post treatment tumors showed a significant reduction in % malignant cells vs. TME (70% to 34% malignant cells vs. 30% to 66% TME) in an immune rich non-fibrotic TME with increased expression of CD8+, Th1+ T cells, cytotoxic T cells, costimulatory ligand, costimulatory receptors, MHCI and MHCII expression, T&B cell traffic, antitumor cytokines and Th2 cells, and a reduction in metastasis and EMT signatures, anti- metastasis factor expression, angiogenesis, matrix and cancer associated fibroblasts. This patient’s tumor had high PD-L1, PD-L2, PD-1 and CTLA5 expression and a high expression of tumor associated M2 macrophages.

The correlation between response with immune cell trafficking in the tumor microenvironment of resected tumors: Stable disease was correlated with a significant decrease in tumor size by histopathologic examination in a patient with rhabdomyosarcoma (**Figure 5**). Histopathologic examination showed high grade pleomorphic rhabdomyosarcoma with tumor free margins, 50% tumor necrosis and increased tumor infiltrating lymphocytes. Comparative evaluation of the molecular functional portraits of pre and post treatment tumors of a patient with advanced rhabdomyosarcoma showed a significant reduction in % malignant cells vs. TME (70% to 34% malignant cells vs. 30% to 66% TME) in an immune rich non-fibrotic TME with increased expression of CD8+, Th1+ T cells, cytotoxic T cells, costimulatory ligand, costimulatory receptors, MHCI and MHCII expression, T&B cell traffic, antitumor cytokines and Th2 cells, and a reduction in metastasis and EMT signatures, anti-metastasis factor expression, angiogenesis, matrix and cancer associated fibroblasts (**Figure 5**). This patient’s tumor had high PD-L1, PD-L2, PD-1 and CTLA5 expression and a high expression of tumor associated M2 macrophages. Currently, the patient is in sustained remission 33 months (2.75 years) from TNT therapy and on no further cancer therapy.

## 4 Discussion

In our Phase 2 study using the TNT regimen, the median PFS was nearly doubled by indirect comparison to that of the Alliance group or a Phase III study using trabectedin alone (median PFS 4.1 months; 15, 23). By indirect comparison, the median OS for both mITT and ITT population was equal or better than first line therapy for soft tissue sarcomas (6). The best responders (PR) were patients with leiomyosarcoma (LMS), liposarcoma (LPS) and undifferentiated pleomorphic sarcoma (UPS). The adverse events were as expected, were mainly related to trabectedin and there were no safety signals reported for the combination TNT regimen. Of note, initial correlation between response and immune cell trafficking in the tumor microenvironment of pre and post treatment tumors of a patient with advanced rhabdomyosarcoma showed stable disease but a significant reduction in % malignant cells vs. TME (70% to 34% malignant cells vs. 30% to 66% TME) in an immune rich non-fibrotic TME and enhanced immune cell trafficking with high expression of PD-L1, PD-L2, CTLA4, CD8+ killer cells, HVEM and tumor associated M2 macrophages in the TME. Currently, the patient is in sustained remission over 2 years from TNT therapy and on no further cancer therapy.

Sarcomas are rare malignant tumors of mesodermal origin with numerous subtypes, genetic mutations and differential sensitivities to cancer therapy (24). The mere fact that there is so much heterogeneity in the pathologic features of sarcomas makes treatment elusive. Standard first line therapy for advanced sarcomas consists of chemotherapy, radiation therapy followed by surgery if the tumor/s become resectable (2, 3; 4, 5; 6–13). The advent of cancer immunotherapy will hopefully provide more treatment options for advanced sarcomas, as immunotherapy drugs are approved by the USFDA and the EMEA/European Commission albeit not yet approved for sarcomas. The challenges of immunotherapy in sarcomas are due to a lack of tumor antigens targetable by vaccines, monoclonal antibodies or chimeric antigen receptor (CAR) therapy and a lack of characterization of the tumor microenvironment (14). A targetable oncogene which is common to all sarcoma subtypes would be ideal.

SARC028 (NCT02301039) evaluated the efficacy and safety of pembrolizumab, a PD-1 inhibitor, in a two-cohort, single-arm, open-label Phase 2 multi-center study. The study showed favorable responses in patients with advanced STS and in patients whose molecular profile showed enhanced tumor infiltrating T cells and tumor associated macrophages expressing PD-L1 (12, 14, 25). Then, Alliance A091401 study (NCT02500797) assessed the efficacy of nivolumab (a PD-1 inhibitor) alone or in combination with ipilimumab (a CTLA4 inhibitor) in advanced sarcomas where the combination regimen was found to be more efficacious in leiomyosarcoma, UPS, myxofibrosarcoma, angiosarcoma, alveolar soft part sarcoma and malignant fibrous histiocytoma. However, even with combinatorial therapy, the median PFS was only 4 months (26).

In recent years, the TMEs of sarcoma histologic subtypes have been characterized (12, 23, 25–28). For instance, Pollack et al. reported that UPS had the highest PD-L1 and PD-1 expression levels when compared to other subtypes and may respond best to immune checkpoint inhibitors. Further, synovial sarcoma expresses NY-ESO-1 and would be suitable for NY-ESO-1 targeted vaccines (25).

There are a number of ways to exploit the immune system against sarcomas including immune checkpoint inhibitors, CAR-T cell therapy, natural killer cell therapy, virotherapy, dendritic cell therapy, and drugs that deplete the tumor microenvironment of M2 growth promoting macrophages (7, 9, 10, 14, 16, 23, 25–31). In this Phase 2 study, we used three of these methods to improve the outcome of patients with advanced sarcomas, namely, Talimogene laherparepvec (an oncolytic virus expressing a cytokine GM-CSF transgene) for maturation of dendritic cells and recruitment of host immune cells into the tumor microenvironment, nivolumab, a PD-1 immune checkpoint inhibitor, and trabectedin (a marine derived alkaloid that favors polarization to M1 macrophages). As a result, the TNT regimen achieved almost a two-fold prolongation of the median PFS (7.8 months) in patients with advanced sarcomas when compared to trabectedin alone (PFS 4.1 months, 15).

Finally, the advent of sophisticated molecular profiling of tumors based on artificial intelligence and bioinformatics (32) is expected to revolutionize the practice of oncology and it is possible that precision medicine will take the place of standard chemotherapy as medical oncologists begin to utilize Artificial Intelligence as a guide for making treatment decisions.

*Limitations.* The main limitations of this study are that it is a non-randomized single center study of a small number of patients with multiple subtypes of sarcoma including one patient with carcinosarcoma, an undifferentiated carcinoma. Indirect comparisons are not definitive statements about the superiority of a treatment regimen over another. The main strength of the study is that the results are promising and hypothesis generating which could be used for planning a Phase 2/3 study.

## 5 Conclusions

This non-randomized phase 2 study met its primary and secondary endpoints which demonstrates the efficacy and safety of the TNT regimen for advanced sarcomas. The treatment regimen is worth being further studied in a randomized phase 3 trial as first- or second- line treatment for patients with advanced sarcomas.

## Data availability statement

The original contributions presented in the study are included in the article/**Supplementary Material**. Further inquiries can be directed to the corresponding author.

## Ethics statement

The studies involving human participants were reviewed and approved by Western IRB. The patients/participants provided their written informed consent to participate in this study.

## Author contributions

Conception and Design: EG, SC, DB; Clinical Investigators: EG, SC, DQ, AM; Data Generation: EG, SC, WT, HC, CV, AA,VC-A; Data Analysis: EG, WT, SC, HC, NO, SM, AA, VC-A, DQ, DB, AM; Manuscript Writing and Approval: EG, WT, SC, HC, CV, AA, NO, SM, DB, VC-A, DQ, AM. All authors contributed to the article and approved the submitted version. 
