# Supplementary material for: Activity of TNT: a phase 2 study using talimogene laherparepvec, nivolumab and trabectedin for previously treated patients with advanced sarcomas (NCT# 03886311)
Source: Front Oncol. 2023 May 10;13:1116937. doi: 10.3389/fonc.2023.1116937 (PMC10206273; doi:10.3389/fonc.2023.1116937)
Supplement: Supplementary file 1 [file DataSheet_1.docx]

**Supplementary Materials.**

**Appendix A. All Adverse Events Related to Study Drugs**

| **Adverse Events Related to Study Therapy, by Grade (n=50)** | | | | | | | | | | | | |
| --- | --- | --- | --- | --- | --- | --- | --- | --- | --- | --- | --- | --- |
| **Adverse Event** | **Related to Trabectedin** | | | | **Related to Nivolumab** | | | | **Related to Talimogene Laherparepvec** | | | |
|  | **1** | **2** | **3** | **4** | **1** | **2** | **3** | **4** | **1** | **2** | **3** | **4** |
| **Blood and lymphatic system disorders** | | | | | | | | | | | | |
| Anemia |  | 1 (2%) | 8 (16%) |  |  |  |  |  |  |  |  |  |
| Eye disorders | | | | | | | | | | | | |
| Blurred vision |  |  |  |  | 1 (2%) |  |  |  | 1 (2%) |  |  |  |
| **Gastrointestinal disorders** | | | | | | | | | | | | |
| Nausea | 9 (18%) | 8 (16%) |  |  |  | 3 (6%) |  |  | 1 (2%) |  |  |  |
| Anorexia | 1 (2%) | 2 (4%) |  |  |  |  |  |  |  |  |  |  |
| Vomiting | 2 (4%) | 4 (8%) |  |  |  | 1 (2%) |  |  |  | 1 (2%) |  |  |
| Constipation | 2 (4%) | 3(6%) |  |  |  |  |  |  |  |  |  |  |
| Diarrhea |  |  |  |  |  | 1 (2%) |  |  |  |  |  |  |
| Bloating | 1 (2%) |  |  |  |  |  |  |  |  |  |  |  |
| Oral sensitivity | 1 (2%) |  |  |  |  |  |  |  |  |  |  |  |
| Diverticulosis |  |  |  |  |  | 1 (2%) |  |  |  |  |  |  |
| Colitis |  |  |  |  |  |  |  |  |  | 1 (2%) |  |  |
| **General disorders and administration site conditions** | | | | | | | | | | | | |
|  |  |  |  |  |  |  |  |  |  |  |  |  |
| Chills |  |  |  |  |  |  |  |  | 4 (8%) | 1 (2%) |  |  |
| Rigors |  |  |  |  |  |  |  |  |  | 1 (2%) |  |  |
| Fatigue | 8 (16%) | 6 (12%) | 2 (4%) |  | 1 (2%) |  |  |  |  |  |  |  |
| Fever |  | 1 (2%) |  |  |  |  |  |  | 5 (10%) | 4 (8%) |  |  |
| Edema, right leg |  |  |  |  |  |  |  |  | 1 (2%) |  |  |  |
| Pain at tumor site |  |  |  |  |  |  |  |  | 1 (2%) |  | 1 (2%) |  |
| Flu like symptoms |  |  |  |  |  |  |  |  | 3 (6%) |  |  |  |
| **Infections and infestations** | | | | | | | | | | | | |
| Cellulitis |  | 1 (2%) |  |  |  |  |  |  | 1 (2%) |  |  |  |
| **Investigations** | | | | | | | | | | | | |
| Alanine aminotransferase increased |  | 2 (4%) | 9 (18%) |  |  |  |  |  |  |  |  |  |
| Aspartate aminotransferase increased |  | 6 (12%) | 3 (6%) |  |  |  |  |  |  |  |  |  |
| Neutrophil count decreased |  |  | 7 (14%) |  |  |  | 1 (2%) |  |  |  |  |  |
| Platelet count decreased |  |  | 4 (8%) | 1 (2%) |  |  |  |  |  |  |  |  |
| CPK increased |  |  | 1 (2%) |  |  |  |  |  |  |  |  |  |
| GGT increased |  |  | 1 (2%) |  |  |  |  |  |  |  |  |  |
| Ejection fraction decreased |  |  | 1 (2%) |  |  |  |  |  |  |  |  |  |
| Parathyroid hormone increased |  |  |  |  | 1 (2%) |  |  |  |  |  |  |  |
| Total bilirubin increased |  | 1 (2%) |  |  |  |  |  |  |  |  |  |  |
| TSH increased | 1 (2%) | 1 (2%) |  |  |  | 2 (4%) |  |  |  |  |  |  |
| Creatinine increased |  | 1 (2%) |  |  |  |  |  |  |  |  |  |  |
| Alkaline phosphatase increased |  | 2 (4%) |  |  |  |  |  |  |  |  |  |  |
| White blood cell decreased |  | 1 (2%) |  |  |  |  |  |  |  |  |  |  |
| T3 decreased |  |  |  |  |  |  | 1 (2%) |  |  |  |  |  |
| **Metabolism and nutrition disorders** | | | | | | | | | | | | |
| Dehydration |  |  | 1 (2%) |  |  |  |  |  |  |  |  |  |
| Hyponatremia |  |  |  |  |  |  | 1 (2%) |  |  |  |  |  |
| Hyperglycemia |  |  |  |  |  | 1 (2%) |  |  |  |  |  |  |
| **Musculoskeletal and connective tissue disorders** | | | | | | | | | | | | |
| Myalgia | 3 (6%) |  |  |  |  |  |  |  | 1 (2%) |  |  |  |
| Arthralgia |  |  |  |  | 1 (2%) |  |  |  |  |  |  |  |
| Joint pain | 1 (2%) |  |  |  |  |  |  |  |  |  |  |  |
| Muscle weakness, general | 1 (2%) |  |  |  |  |  |  |  |  |  |  |  |
| Bone pain |  |  |  |  |  |  |  |  | 1 (2%) |  |  |  |
| Pain, RLE |  |  |  |  |  |  |  |  | 1 (2%) |  |  |  |
| Pain, LLE |  |  |  |  |  |  |  |  |  | 2 (4%) |  |  |
| **Nervous system disorders** | | | | | | | | | | | | |
| Headache |  |  |  |  |  |  |  |  | 1 (2%) |  |  |  |
| Paresthesia |  |  |  |  |  |  |  |  |  | 1 (2%) |  |  |
| **Respiratory, thoracic and mediastinal disorders** | | | | | | | | | | | | |
| Cough |  |  |  |  |  |  |  |  |  | 1 (2%) |  |  |
| Pneumonitis |  |  |  |  |  | 1 (2%) |  |  |  |  |  |  |
| **Skin and subcutaneous tissue disorders** | | | | | | | | | | | | |
| Rashes |  |  |  |  |  |  |  |  | 1 (2%) |  |  |  |
| Erythema |  |  |  |  |  |  |  |  | 1 (2%) |  |  |  |

**Appendix B. All Adverse Events Not Related to Study Drugs(n=50)**

| **Adverse Events Unrelated to Study Therapy, by Grade (n=50)** | | | | | |
| --- | --- | --- | --- | --- | --- |
| **Adverse Event** | **Grade** | | | | |
|  | **1** | **2** | **3** | **4** | **5** |
| **Blood and lymphatic system disorders** | | | | | |
| Anemia |  | 2 (4%) | 6 (12%) |  |  |
| Cardiac disorders | | | | | |
| Atrial fibrillation |  | 1 (2%) |  |  |  |
| Tachycardia |  | 2 (4%) |  |  |  |
| **Endocrine disorders** | | | | | |
| Adrenal insufficiency |  |  | 1 (2%) |  |  |
| Eye disorders | | | | | |
| Right eye pruritus | 1 (2%) |  |  |  |  |
| Blurry vision | 1 (2%) |  |  |  |  |
| Nystagmus | 1 (2%) |  |  |  |  |
| **Gastrointestinal disorders** | | | | | |
| Toothache | 1 (2%) |  |  |  |  |
| Nausea | 7 (14%) | 4 (8%) |  |  |  |
| Vomiting | 4 (8%) | 3 (6%) |  |  |  |
| Abdominal pain | 1 (2%) | 5 (10%) | 2 (4%) |  |  |
| Constipation | 2 (4%) | 4 (8%) |  |  |  |
| Diarrhea | 2 (4%) | 3 (6%) |  |  |  |
| Abdominal distension | 5 (10%) | 3 (6%) |  |  |  |
| Anorexia | 2 (4%) |  | 1 (2%) |  |  |
| Heartburn | 2 (4%) |  |  |  |  |
| Obstipation |  | 1 (2%) |  |  |  |
| Flatulence |  | 1 (2%) |  |  |  |
| Bloating | 1 (2%) |  |  |  |  |
| Dyspepsia |  | 1 (2%) |  |  |  |
| Profuse rectal bleeding |  |  | 1 (2%) |  |  |
| Gastroesophageal Reflux Disease | 1 (2%) |  |  |  |  |
| **General disorders and administration site conditions** | | | | | |
| Increased tenderness around tumor site |  | 1 (2%) |  |  |  |
| Tenderness on port site | 1 (2%) |  |  |  |  |
| Swelling of tumor site |  | 1 (2%) |  |  |  |
| Edema, lower left extremity | 1 (2%) |  |  |  |  |
| Fever | 5 (10%) | 1 (2%) |  |  |  |
| Chills | 1 (2%) | 1 (2%) |  |  |  |
| Fatigue | 7 (14%) | 7 (14%) |  |  |  |
| Edema, right lower extremity | 1 (2%) |  |  |  |  |
| Anasarca |  | 1 (2%) |  |  |  |
| Edema Bilateral Lower extremities |  | 3 (6%) |  |  |  |
| Lateral left chest wall mass | 1 (2%) |  |  |  |  |
| Pain at tumor site |  | 1 (2%) |  |  |  |
| Post op pain |  |  | 1 (2%) |  |  |
| Breast pain | 1 (2%) |  |  |  |  |
| Bilateral pedal edema | 1 (2%) | 1 (2%) |  |  |  |
| Non-cardiac chest pain | 1 (2%) | 2 (4%) |  |  |  |
| Right leg edema | 1 (2%) |  |  |  |  |
| Right leg pain |  | 1 (2%) |  |  |  |
| Left pleuritic pain | 1 (2%) |  |  |  |  |
| Asthenia |  | 1 (2%) |  |  |  |
| Rigors | 1 (2%) |  |  |  |  |
| Pain at radiation site |  | 1 (2%) |  |  |  |
| Left neck tumor swelling |  | 1 (2%) |  |  |  |
| New osseous lesions in thoracic spine |  | 1 (2%) |  |  |  |
| Difficulty swallowing | 1 (2%) |  |  |  |  |
| Foul odor in urine | 1 (2%) |  |  |  |  |
| Generalized pain |  | 1 (2%) |  |  |  |
| New upper back lesions |  | 1 (2%) |  |  |  |
| Edema, left upper extremity |  | 1 (2%) |  |  |  |
| **Infections and infestations** | | | | | |
| Urinary tract infection | 2 (4%) | 2 (4%) |  |  |  |
| Otitis media | 1 (2%) |  |  |  |  |
| Sepsis | 1 (2%) |  |  |  |  |
| Cellulitis at port site | 1 (2%) |  |  |  |  |
| **Investigations** | | | | | |
| Neutrophil count decreased | 1 (2%) | 1 (2%) |  |  |  |
| Platelet count decreased |  | 1 (2%) |  |  |  |
| White blood cell decreased | 1 (2%) |  |  |  |  |
| Creatinine increased |  | 1 (2%) |  |  |  |
| TSH increased | 2 (4%) |  |  |  |  |
| **Metabolism and nutrition disorders** | | | | | |
| Hypokalemia | 2 (4%) | 1 (2%) | 1 (2%) |  |  |
| Hypoalbuminemia |  | 1 (2%) | 1 (2%) |  |  |
| Hypocalcemia |  | 2 (4%) |  |  |  |
| Vitamin B12 deficiency | 1 (2%) |  |  |  |  |
| Vitamin D deficiency | 1 (2%) |  |  |  |  |
| Acute respiratory alkalosis |  | 1 (2%) |  |  |  |
| Hyponatremia | 1 (2%) | 1 (2%) |  |  |  |
| Hypophosphatemia |  |  | 1 (2%) |  |  |
| Dehydration |  | 2 (4%) |  |  |  |
| Poor fluid intake | 1 (2%) |  |  |  |  |
| **Musculoskeletal and connective tissue disorders** | | | | | |
| Muscle spasm, LLE |  | 1 (2%) |  |  |  |
| Hip pain | 2 (4%) | 1 (2%) |  |  |  |
| Pain on bilateral lower extremities | 1 (2%) |  |  |  |  |
| Pain, lower back | 1 (2%) | 1 (2%) | 1 (2%) |  |  |
| Knee Pain | 1 (2%) |  |  |  |  |
| Sternal pain | 1 (2%) |  |  |  |  |
| Leg pain |  |  | 1 (2%) |  |  |
| Arm pain | 1 (2%) |  |  |  |  |
| Back pain | 1 (2%) | 2 (4%) | 1 (2%) |  |  |
| Chest wall pain | 1 (2%) |  | 1 (2%) |  |  |
| Muscle tightness | 1 (2%) |  |  |  |  |
| Myalgia | 1 (2%) |  |  |  |  |
| Pain on right lower extremity | 2 (4%) | 1 (2%) | 1 (2%) |  |  |
| Hyperostosis frontalis interna | 1 (2%) |  |  |  |  |
| Shoulder pain |  | 1 (2%) |  |  |  |
| Left leg pain |  | 1 (2%) |  |  |  |
| Neck and shoulder pain |  | 1 (2%) |  |  |  |
| Left flank pain |  |  | 1 (2%) |  |  |
| Right leg pain | 1 (2%) | 1 (2%) |  |  |  |
| Left groin pain |  | 1 (2%) |  |  |  |
| Arthralgia in left knee |  | 1 (2%) |  |  |  |
| Groin pain |  | 2 (4%) |  |  |  |
| Right flank pain |  | 1 (2%) |  |  |  |
| Right thigh pain |  | 1 (2%) |  |  |  |
| Rib pain | 1 (2%) |  |  |  |  |
| Weakness on bilateral lower extremities | 1 (2%) |  |  |  |  |
| **Nervous system disorders** | | | | | |
| Headache | 5 (10%) | 1 (2%) |  |  |  |
| Peripheral neuropathy, bilateral hands | 1 (2%) |  |  |  |  |
| Encephalopathy |  | 1 (2%) |  |  |  |
| Dizziness |  | 1 (2%) |  |  |  |
| Peripheral neuropathy, lower extremities | 1 (2%) | 1 (2%) |  |  |  |
| Syncope | 1 (2%) |  |  |  |  |
| **Psychiatric disorders** | | | | | |
| Insomnia | 6 (12%) | 2 (4%) |  |  |  |
| Anxiety | 1 (2%) | 3 (6%) |  |  |  |
| Depression | 1 (2%) |  |  |  |  |
| Sleep disturbance | 1 (2%) |  |  |  |  |
| **Renal and urinary disorders** | | | | | |
| Dysuria |  | 1 (2%) |  |  |  |
| Urinary obstruction |  |  | 1 (2%) |  |  |
| **Respiratory, thoracic, and mediastinal disorders** | | | | | |
| Dyspnea | 4 (8%) | 2 (4%) | 4 (8%) |  |  |
| Nasal congestion | 1 (2%) |  |  |  |  |
| Bronchitis |  |  | 1 (2%) |  |  |
| Wheezing |  | 1 (2%) |  |  |  |
| Cough | 3 (6%) | 1 (2%) |  |  |  |
| Hiccups |  | 1 (2%) |  |  |  |
| Hoarseness | 1 (2%) |  |  |  |  |
| Acute respiratory failure with hypoxia |  |  | 1 (2%) |  |  |
| Hypoxemia |  | 1 (2%) | 1 (2%) |  |  |
| Post obstructive pneumonia |  |  | 1 (2%) |  |  |
| Respiratory distress |  | 1 (2%) | 1 (2%) |  |  |
| Epistaxis | 1 (2%) |  |  |  |  |
| Pneumonia |  | 1 (2%) |  |  |  |
| Hemoptysis | 1 (2%) | 2 (4%) | 1 (2%) |  |  |
| **Skin and subcutaneous tissue disorders** | | | | | |
| Vaginal pruritus |  | 1 (2%) |  |  |  |
| Vulvae pruritus |  | 1 (2%) |  |  |  |
| Pruritus on right lower extremities | 1 (2%) |  |  |  |  |
| Erythema on port site | 1 (2%) |  |  |  |  |
| Shingles |  | 1 (2%) |  |  |  |
| Head and neck pruritus |  | 1 (2%) |  |  |  |
| Erythema near site of radiation | 1 (2%) |  |  |  |  |
| Pruritus on left calf near radiation site | 1 (2%) |  |  |  |  |
| Erythema on tumor site | 1 (2%) |  |  |  |  |
| **Vascular disorders** | | | | | |
| Deep vein thrombosis, right lower extremity |  | 1 (2%) |  |  |  |
| Deep vein thrombosis, right neck | 1 (2%) |  |  |  |  |
| Hypotension |  | 2 (4%) |  |  |  |
| Deep vein thrombosis, right knee |  | 1 (2%) |  |  |  |
| Facial flushing | 1 (2%) |  |  |  |  |
| Deep vein thrombosis, left thigh | 1 (2%) |  |  |  |  |

**Appendix C. Listing of Deaths**

| **Patient Number** | **Time (Days) between Last Dose and Death** | **Time (Months) between Last Dose and Death** |
| --- | --- | --- |
| 1 | 60 | 2.0 |
| 2 | 47 | 1.5 |
| 3 | 273 | 9.0 |
| 4 | 135 | 4.4 |
| 5 | 312 | 10.2 |
| 6 | 2 | 0.1 |
| 7 | 162 | 5.3 |
| 8 | 347 | 11.4 |
| 9 | 588 | 19.3 |
| 10 | 406 | 13.3 |
| 11 | 208 | 6.8 |
| 12 | 60 | 2.0 |
| 13 | 65 | 2.1 |
| 14 | 53 | 1.7 |
| 15 | 349 | 11.5 |
| 16 | 102 | 3.3 |
| 17 | 8 | 0.3 |
| 18 | 31 | 1.0 |
| 19 | 303 | 9.9 |
| 20 | 262 | 8.6 |
| 21 | 6 | 0.2 |
| 22 | 15 | 0.5 |
| 23 | 154 | 5.1 |
| 24 | 7 | 0.2 |
| 25 | 16 | 0.5 |
| 26 | 65 | 2.1 |
| 27 | 82 | 2.7 |
| 28 | 93 | 3.1 |
| 29 | 105 | 3.4 |

**Appendix D. Listing of Patients Who Withdrew from Study Due to Adverse Event**

| **No. of Patients** | **Time (Days) between Last Dose and Withdrawal** | **Adverse Event** | **Attributable to drug (Y/N)** |
| --- | --- | --- | --- |
| 1 | 103 | Recurrent colitis | Y |
| 2 | 11 | Severe nausea and vomiting | Y |
| 3 | 14 | Persistent pain | N |
| 4 | 54 | Persistent pain | N |
| 5 | 22 | Fatigue, abdominal pain, poor appetite and weight loss | Y |
| 6 | 70 | Sepsis | N |

**Appendix E. Listing of Patients Who were Lost to Follow-up**

| **No. of Patients** | **Days between Date of Last Dose and Date of Last Contact** | **Clinical Status** |
| --- | --- | --- |
| 1 | 505 | SD |
| 2 | 26 | SD |
| 3 | 355 | SD |
| 4 | 42 | SD |
| 5 | 27 | NE |
| 6 | 37 | PD |
